# E3 ubiquitin ligase Grail promotes hepatic steatosis through Sirt1 inhibition

**DOI:** 10.1038/s41419-021-03608-9

**Published:** 2021-03-26

**Authors:** Pei-Yao Liu, Cheng-Cheung Chen, Chia-Ying Chin, Te-Jung Liu, Wen-Chiuan Tsai, Jian-Liang Chou, Chuan-Yu Huang, Yu-Guang Chen, Ying-Chuan Chen

**Affiliations:** 1 Department of Physiology & Biophysics, National Defense Medical Center, Taipei, 114 Taiwan; 2 Institute of Preventive Medicine, National Defense Medical Center, New Taipei City, Taiwan; 3 Graduate Institute of Life Sciences, National Defense Medical Center, Taipei, 114 Taiwan; 4 Department of Physical Medicine and Rehabilitation, Tri-Service General Hospital, Taipei, 114 Taiwan; 5 Department of Physical Medicine and Rehabilitation, School of Medicine, National Defense Medical Center, Taipei, 114 Taiwan; 6 Department of Physical Medicine and Rehabilitation, Taoyuan Armed Force General Hospital, Taoyuan, 114 Taiwan; 7 Department of Pathology, Tri-Service General Hospital, National Defense Medical Center, Taipei, 114 Taiwan; 8 Graduate Institute of Pathology and Parasitology, National Defense Medical Center, Taipei, 114 Taiwan; 9 Instrument Center, Department of Research and Development, National Defense Medical Center, Taipei, 114 Taiwan; 10 Division of Hematology/Oncology, Department of Internal Medicine, Tri-Service General Hospital, National Defense Medical Center, Taipei, 114 Taiwan; 11 Cancer Institute, University College London, London, UK

**Keywords:** Cell signalling, Metabolic disorders

## Abstract

In obese adults, nonalcoholic fatty liver disease (NAFLD) is accompanied by multiple metabolic dysfunctions. Although upregulated hepatic fatty acid synthesis has been identified as a crucial mediator of NAFLD development, the underlying mechanisms are yet to be elucidated. In this study, we reported upregulated expression of gene related to anergy in lymphocytes (*GRAIL*) in the livers of humans and mice with hepatic steatosis. Grail ablation markedly alleviated the high-fat diet-induced hepatic fat accumulation and expression of genes related to the lipid metabolism, in vitro and in vivo. Conversely, overexpression of *GRAIL* exacerbated lipid accumulation and enhanced the expression of lipid metabolic genes in mice and liver cells. Our results demonstrated that Grail regulated the lipid accumulation in hepatic steatosis via interaction with sirtuin 1. Thus, Grail poses as a significant molecular regulator in the development of NAFLD.

## Introduction

The common symptoms of hepatic steatosis or fatty liver, such as chronic inflammation and steatosis, may result in nonalcoholic steatohepatitis (NASH), cirrhosis, and even liver cancer, if unattended^1–3^. Nonalcoholic fatty liver disease (NAFLD) is a common event in all chronic liver diseases. A majority of the studies have proposed 25–45% prevalence of NAFLD^4,5^. It has been shown that obesity, cardiovascular disease, and diabetes are closely related to NAFLD^6^. NAFLD is caused by excessive accumulation of fat in the liver resulting from increased hepatic de novo lipid synthesis and reduced beta (β)-oxidation of fatty acids^7,8^. Although studies have reported the involvement of signaling pathways such as the sirtuin 1 (Sirt1)-signaling pathway in the progression of hepatitis steatosis^9,10^, the detail molecular mechanisms are yet to be deciphered.

Gene related to anergy in lymphocytes (Grail) is a type I transmembrane protein, which participates in the endocytic system and has a crucial role in T cell anergy^11,12^. Grail also enhances the degradation of proteins involved in CD4^+^ T cell activation, such as the cytoskeletal proteins and the antigen-presenting cell (APC) receptors^13–17^. Grail remains associated with the adipocyte differentiation process, and the deletion of Grail protects from high-fat diet (HFD)-induced obesity^18^. Grail has been identified as one of the targets of signal transducer and activator of transcription 3 (*STAT3*) and can eventually reduce the alternative activation of macrophages^19^. Above all, these studies demonstrated the potential role of Grail in regulating energy homeostasis and obesity-induced inflammation. However, its roles in hepatic lipid accumulation and metabolism have not been elucidated.

In this study, we used adeno-associated virus serotype 8 (AAV8)-mediated Grail overexpression in HFD-fed mice and *GRAIL* knockout (KO) mice to investigate the role of Grail in NAFLD development. We also demonstrated the role of Grail in hepatic steatosis and the molecular mechanisms underlying targeted degradation of Grail-bound Sirt1. These findings would help identify targets specific for NAFLD treatment.

## Results

### Grail is upregulated in the livers of obese mice and palmitate acid-treated liver cells

**Fig. 1:**
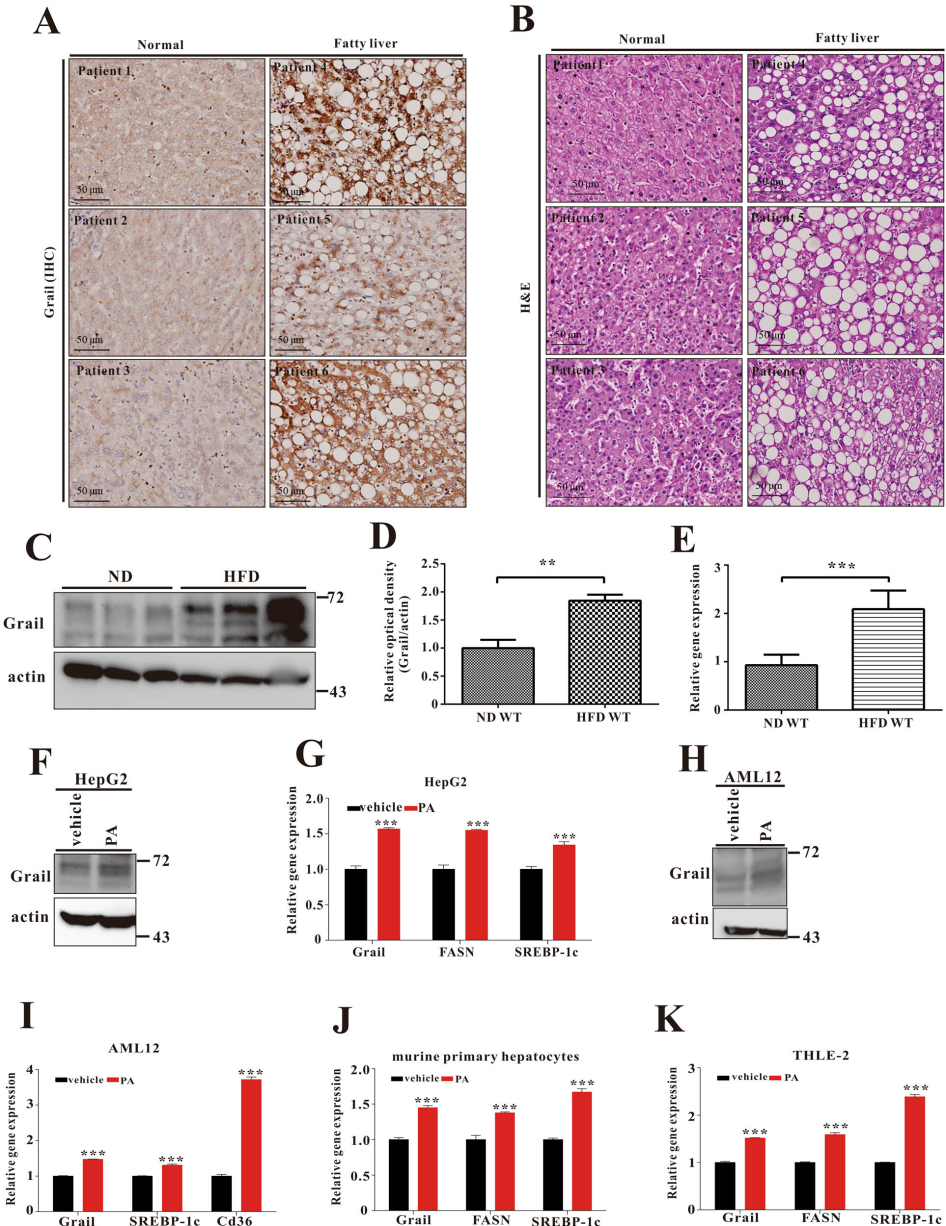
GRAIL expression is upregulated in the livers of patients with nonalcoholic fatty liver disease (NAFLD) and obese mice. **A, B** Histological images of liver sections from NAFLD or normal patients stained using Grail antibody and hematoxylin and eosin stain (scale bar, 50 μm). **C–E** Grail protein and mRNA expression in the liver of mice subjected to high-fat diet (HFD) and in controls fed on normal chow diet (ND). **F, G** HepG2 (**H, I**), AML12 (**J**), murine primary hepatocytes (**K**), and THLE-2 cells were stimulated with palmitic acid (800 μM) for 24 h and GRAIL expression was examined by immunoblotting. The mRNA levels of *GRAIL*, fatty acid synthase (*FASN*), sterol regulatory element-binding protein 1c (*SREBP-1c*), and *CD36* were measured by real-time quantitative polymerase chain reaction (RT-qPCR). The data are presented as mean ± standard deviation (SD). **P < 0.01; ***P < 0.001, Student’s *t-*test.

According to previous studies, Grail can affect adipocyte differentiation and lipid accumulation^18^. Here, we were interested in the expression and function of Grail with NAFLD development. First, we analyzed the Grail expression of liver samples from with NAFLD and normal patients by immunohistochemistry and found that expression of Grail was obviously increased in fatty liver patients (Fig. 1A, B). Next, we observed the Grail expression of liver from the mice feeding with normal diet (ND) and 60% HFD (D12492) through immunoblotting and mRNA analysis. The liver of HFD mice showed higher protein expression (Fig. 1C) and result was quantitated (Fig. 1D). The pattern of mRNA expression was the same (Fig. 1E). Furthermore, we focused on the hepatocyte of Grail expression after fatty acid treatment. Consistent with the findings in the liver of human and mice, both the mRNA and protein expression of Grail significantly increased in the murine primary hepatocytes (Fig. 1J), hepatocellular carcinoma cell line as HepG2 (Fig. 1F, G), murine liver cell line as AML12 (Fig. 1H, I), and human liver cell line as THLE-2 (Fig. 1K).

### *GRAIL* deletion ameliorates hepatic steatosis

**Fig. 2:**
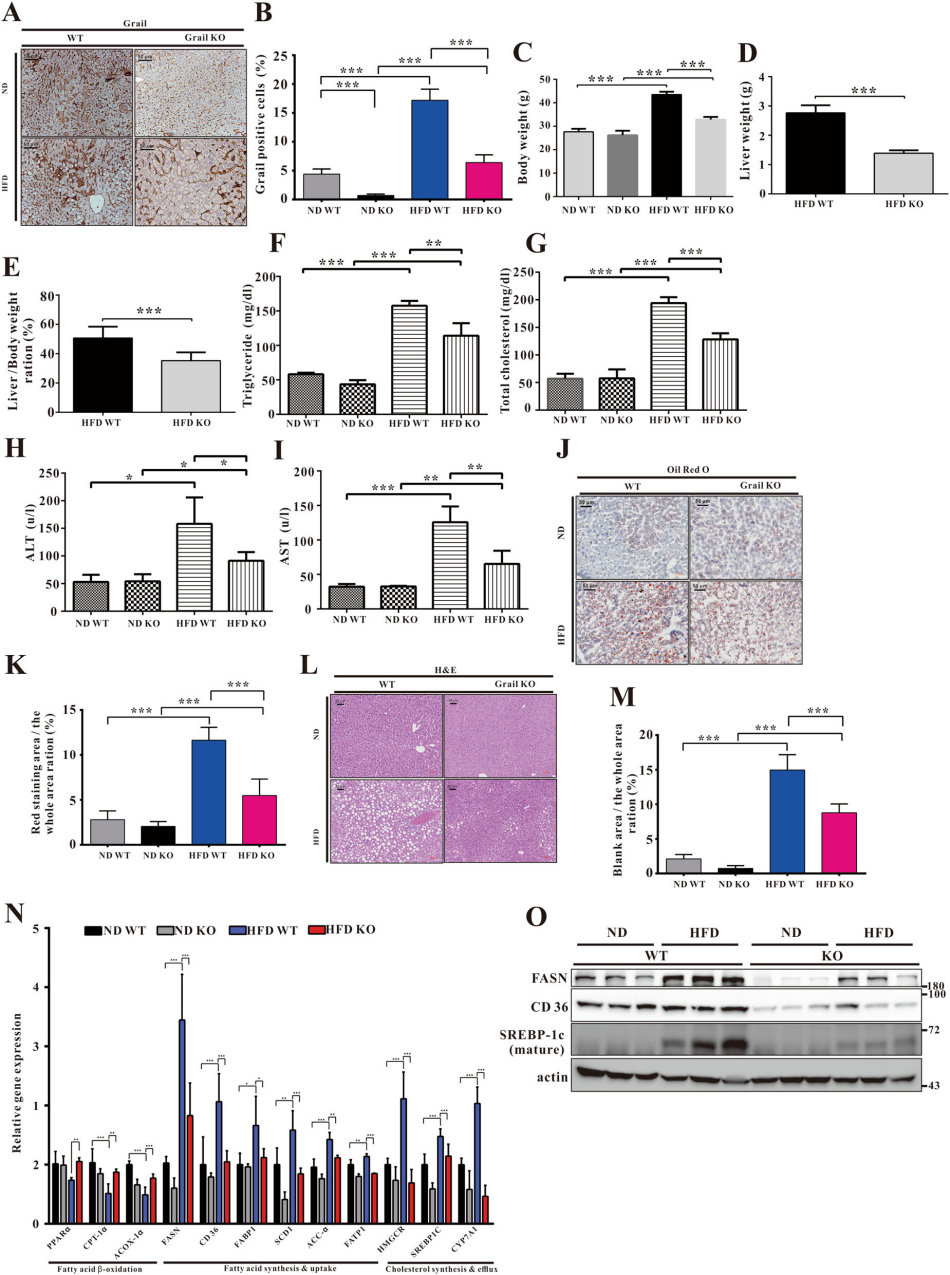
GRAIL deficiency attenuates high-fat diet (HFD)-induced hepatic steatosis and obesity. **A, B** Histological images of liver tissues after Grail staining in the indicated groups. **C** Wild-type (WT) and *GRAIL* knockout (KO) mice were fed normal chow diet (ND) (*n* = 5) or HFD (*n* = 5) for 8 weeks, and their body weights were determined. **D, E** Liver weight (*n* = 5) and Liver to body weight ratio (*n* = 5) of the WT and *GRAIL* KO mice after an 8-week ND or HFD treatment. **F–I** Analysis of serum triglyceride (TG), total cholesterol (TC), alanine aminotransferase (ALT), and aspartate aminotransferase (AST) in the indicated groups after ND or HFD treatment (*n* = 5/group). **J–M** Histological images of liver tissues after hematoxylin and eosin (HE) staining and oil red O staining in the indicated groups. **N** The mRNA expressions of genes related to cholesterol synthesis and efflux and fatty acid uptake, synthesis, and beta-oxidation in the liver samples of WT and *GRAIL* KO mice after ND or HFD treatment (*n* = 5–6/group), determined using real-time quantitative polymerase chain reaction (RT-qPCR). The data are presented as mean ± standard deviation (SD). **O** Fatty acid synthase (FASN), CD36, and sterol regulatory element-binding protein 1c (SREBP-1c) (mature form) in liver samples of WT and *GRAIL* KO mice fed with ND or HFD were detected with immunoblotting. One-way ANOVA with Newman-Keuls post hoc test or Student’s *t-*test are used to evaluate the statistical significance. *P < 0.05; **P < 0.01; ***P < 0.001.

To elucidate the physiological role of Grail in NAFLD development in vivo, we used a *Grail* KO mouse model to determine the effects of Grail on NAFLD. To that end, the WT and Grail KO mice were fed ND or 60% HFD (D12492) for 8 weeks. We analyzed the Grail expression of liver samples from with ND and HFD-fed mice by immunohistochemistry and found that expression of Grail was obviously lowered in Grail KO mice (Fig. 2A, B). Furthermore, measurement of the mice body weight after ablation of Grail and feeding on HFD or ND, we observed lower weight gain in *GRAIL* KO mice after HFD feeding (Fig. 2C). In terms of weight of the liver organs, no significant difference between the wild-type (WT) and KO in ND fed mice was observed. However, when fed with HFD, the weight of the liver was statistically higher in the WT mice (Fig. 2D, E), compared to the KO mice. The results indicated that the *GRAIL KO* mice were resistant to HFD-induced liver weight gain. Analysis of the biochemical parameters, including serum triglycerides (TG) and total cholesterol after HFD and ND feeding, revealed both serum TG and the total cholesterol to be significantly increased in the WT after HFD compared to ND. The increases were significantly reversed in the HFD-fed KO mice (Fig. 2F, G). Given the correlation between hepatocellular injury and increased serum concentration of alanine aminotransferase (ALT) and aspartate aminotransferase (AST)^20^, we measured serum ALT and AST as indicators of the liver function and observed significantly increased serum levels of both ALT and AST in HFD-fed WT mice. However, the increases were reversed in the HFD-fed KO mice (Fig. 2H, I). Hematoxylin and eosin staining (HE) and oil red O staining of the liver section showed severe lipid accumulation in the HFD-fed WT mice, while the HFD-fed KO mice exhibited minimal hepatic lipid accumulation (Fig. 2J–M). The mRNA levels of genes related to cholesterol synthesis and efflux (3-hydroxy-3-methylglutaryl CoA reductase (*HMGCR*), sterol regulatory element-binding protein 1c (SREBP-1c), and cholesterol 7 alpha-hydroxylase (*CYP7A*)) and fatty acid uptake and synthesis (CD36, fatty acid transport protein 1 (*FATP1*), fatty acid-binding protein 1(*FABP1*), fatty acid synthase (*FASN*), stearoyl-CoA desaturase-1 (*SCD1*), and acetyl CoA carboxylase 1 (*ACC1*)) were attenuated in HFD-fed KO mice. Conversely, mRNA levels of genes related to fatty acid β-oxidation (peroxisome proliferator-activated receptor alpha (*PPARA*), carnitine palmitoyltransferase 1 (*CPT1*) and peroxisomal acyl-coenzyme A oxidase 1 (*ACOX1*)) were increased in HFD-fed KO mice (Fig. 2N). In-line with this, the mRNA levels of genes related to cholesterol synthesis, cholesterol efflux, fatty acid uptake, and fatty acid synthesis were decreased and that for fatty acid β-oxidation were augmented in the primary hepatocytes derived from HFD-fed KO mice (Fig. S1). Congruent with the expectations, the protein levels of FASN, CD36, and mature form of SREBP-1c decreased in the HFDKO mice (Fig. 2O). In summary, these results revealed the resistance of *GRAIL* KO mice to HFD-induced hepatic steatosis.

### AAV8-mediated *GRAIL* overexpression exacerbates HFD-induced hepatic steatosis

**Fig. 3:**
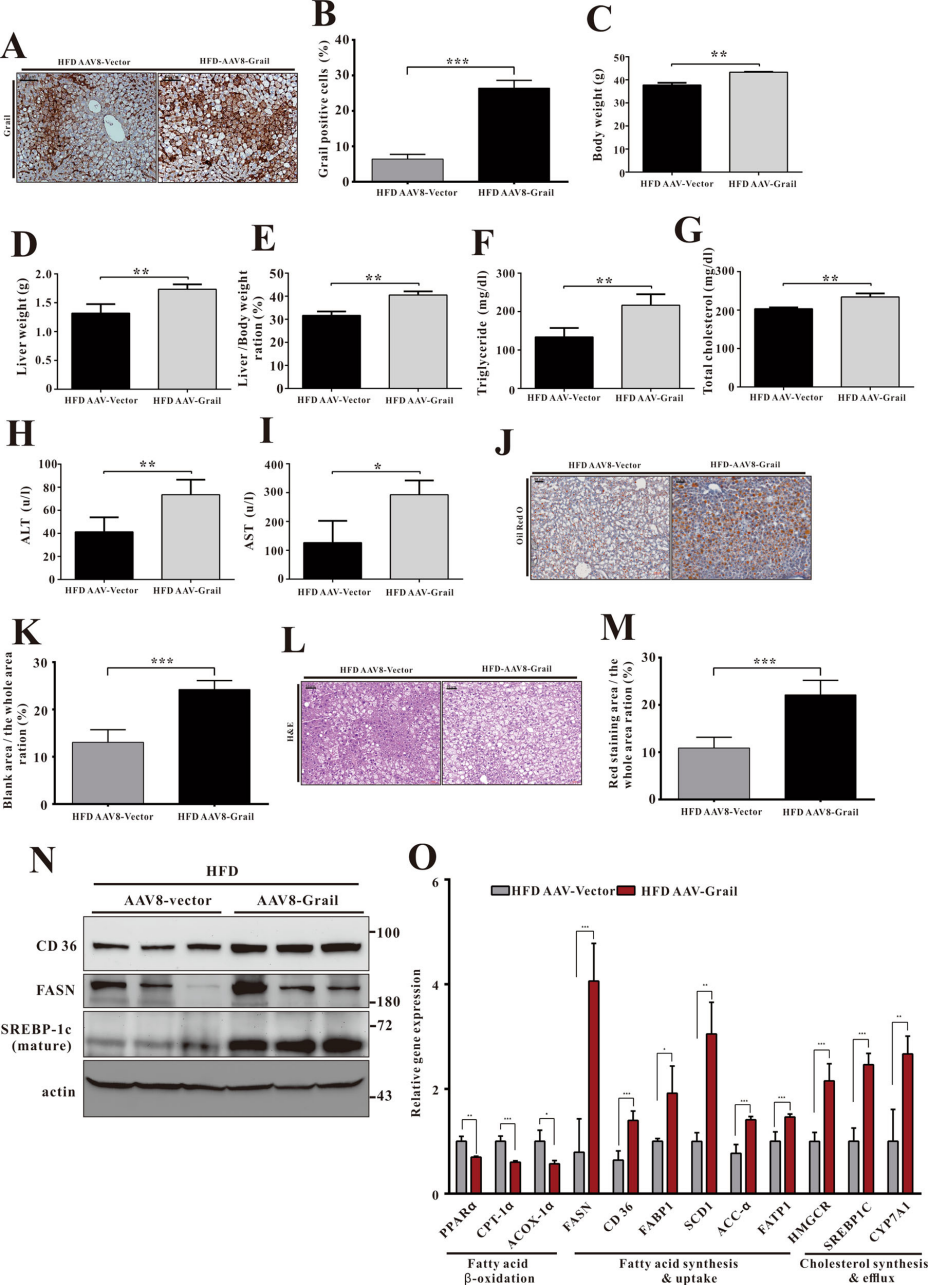
GRAIL overexpression aggravates hepatic steatosis. **A, B** Histological images of liver tissues after Grail staining in the indicated groups. **C** Body weights, **D** Liver weight, and **E** Liver to body weight ratio of adeno-associated virus serotype 8 (AAV8)-vector and AAV8-Grail mice after 8 weeks of high-fat diet (HFD) feeding. **F–I** Changes of triglyceride (TG), total cholesterol (TC), alanine aminotransferase (ALT), and aspartate aminotransferase (AST) in the serum obtained from indicated groups after HFD treatment. **J–M** Histological images of liver tissues after hematoxylin and eosin (HE) staining and oil red O staining in the indicated groups. **N** The fatty acid synthase (FASN), CD36, and matured form of sterol regulatory element-binding protein 1c (SREBP-1c) in liver samples of AAV8-vector and AAV8-Grail mice fed with HFD were detected with immunoblotting. **O** The mRNA expressions of genes related to cholesterol synthesis and efflux and fatty acid uptake, synthesis, and beta-oxidation in the liver samples of AAV8-vector and AAV8-Grail mice after HFD treatment, determined using real-time quantitative polymerase chain reaction (RT-qPCR). The data are presented as mean ± SD. Student’s *t-*test are used to evaluate the statistical significance. *P < 0.05; **P < 0.01; ***P < 0.001.

To further verify the regulatory function of Grail in hepatic steatosis, we employed the AAV8 for overexpression of *GRAIL* in the liver. We analyzed the Grail expression of liver samples from 60% HFD (D12492) fed AAV8-Vector or AAV8-Grail mice by immunohistochemistry and found that expression of Grail was obviously increased in AAV8-Grail mice (Fig. 3A, B). In contrast to the observations in *GRAIL* KO mice, the body weight, weight gain, and liver weight significantly increased in response to HFD treatment in the AAV8-Grail mice compared to the controls (Fig. 3C–E). Increase in the serum ALT, AST, TG, and cholesterol levels were also evident in AAV8-Grail mice subjected to HFD feeding (Fig. 3F–I). Moreover, the HFD-fed AAV8-Grail mice exhibited excessive accumulation of lipids in their livers (Fig. 3J–M). Congruent with the expectations, increase in the protein levels of FASN, CD36, and mature form of SREBP-1c were observed in HFD-fed AAV8-Grail mice (Fig. 3N). Consistently, the mRNA levels of genes for cholesterol efflux and synthesis and fatty acid uptake and synthesis were significantly upregulated, and that for fatty acid β-oxidation were downregulated in HFD-fed AAV8-Grail mice compared to the controls (Fig. 3O). The evidences reconfirmed the regulatory role of Grail in HFD-induced hepatic steatosis.

### Loss of *GRAIL* expression reduces lipid accumulation

**Fig. 4:**
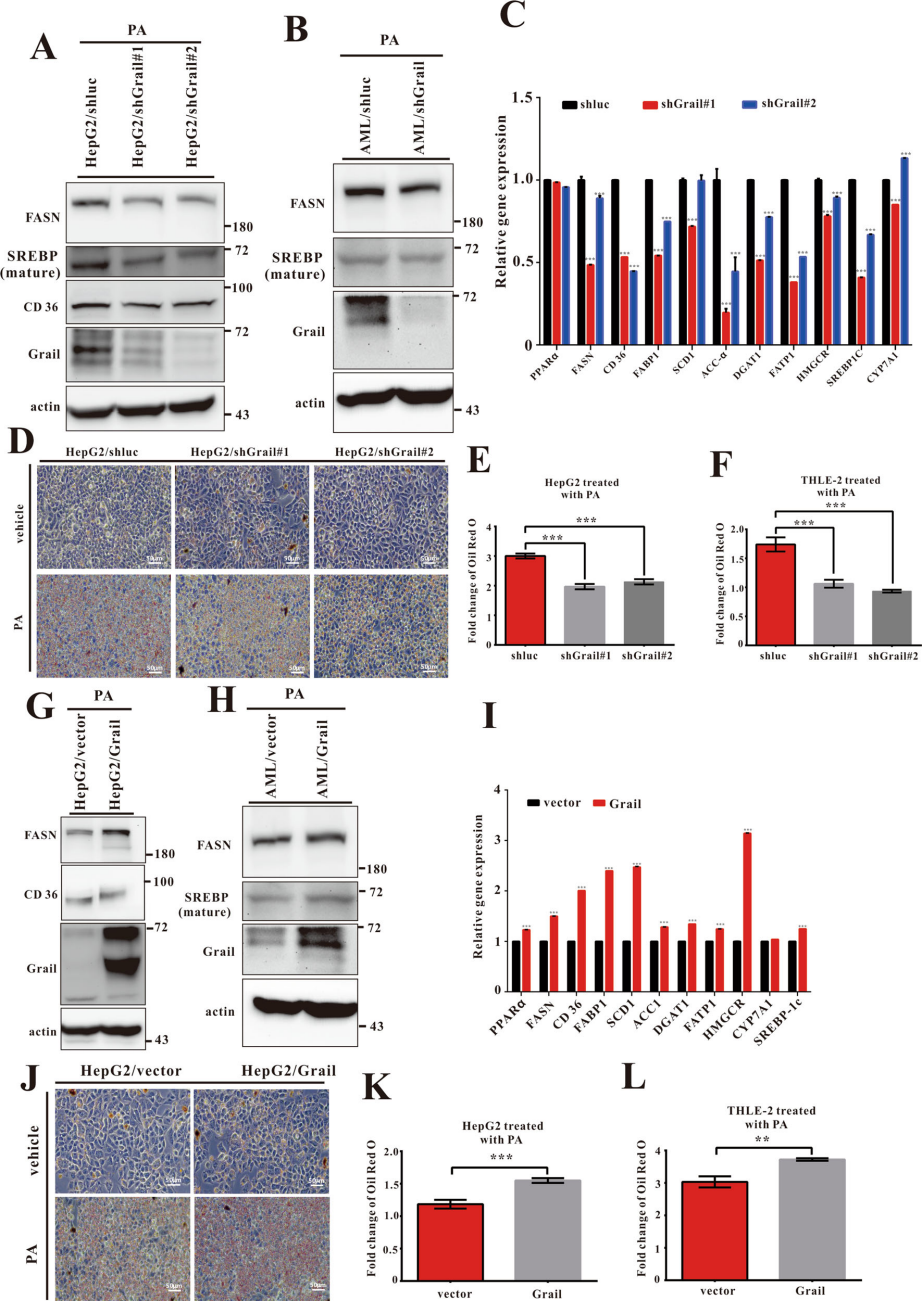
GRAIL promotes lipid synthesis, uptake, and accumulation in vitro. **A, B** Immunoblots of Grail, fatty acid synthase (FASN), CD36, and mature form of sterol regulatory element-binding protein 1c (SREBP-1c) in indicated cell lines. **C** The mRNA expressions of genes related to cholesterol synthesis and efflux and fatty acid uptake and synthesis in indicated samples. **D, E** Lipid accumulations displayed by oil red O staining in HepG2/shluc and HepG2/shGrail or **F** in THLE-2/shluc and THLE-2/shGrail cell lines, and subjected to PA (800 μM) or vehicle control administration for 24 h. **G, H** Immunoblots of Grail, FASN, CD36, and mature form of SREBP-1c in HepG2/Grail or AML/Grail cell lines. **I** The mRNA expressions of genes related to cholesterol synthesis and efflux and fatty acid uptake and synthesis were detected in HepG2/vector and HepG2/Grail cells. Lipid accumulations displayed by oil red O staining **J, K** in HepG2/vector and HepG2/Grail or **L** in THLE-2/vector and THLE-2/Grail cell lines after 24 h PA (800 μM) treatment. The data are presented as mean ± SD. Student’s *t-*test are used to evaluate the statistical significance. **P < 0.01; ***P < 0.001.

We further characterized the relationship between Grail and hepatic lipid accumulation in vitro, by silencing the expression of *GRAIL* in HepG2 (HepG2/shGrail) cells and confirming its downregulation (Fig. 4A and Fig. S2). Measurement of the mRNA levels of genes related to cholesterol synthesis, cholesterol efflux, fatty acid uptake, and fatty acid synthesis upon treatment with palmitic acid (PA) revealed that the mRNA levels of lipid metabolism-related genes were attenuated in the PA-treated HepG2/shGrail cells compared to the controls (Fig. 4C). Consistently, the mRNA levels of lipid metabolism-related genes also increased in the AML12/Grail cells compared to the controls (Fig. S3A). Ablation of Grail resulted in lowered levels of the mature form of SREBP-1c (Fig. 4A). Likewise, the protein levels of FASN and CD36 were also reduced in the PA-treated HepG2/shGrail cells (Fig. 4A). Similar results were observed in the AML12/shGrail cells (Fig. 4B). Oil Red O staining showed reduced lipid accumulation capacity in *GRAIL* knockdown cells compared to the controls exposed to PA treatment (Fig. 4D, E). Consistent with the findings in the HepG2/shGrail cells, lipid accumulation was decreased in the THLE-2/shGrail, Huh7/shGrail, and AML12/shGrail cells compared to the controls (Fig. 4F and S4). Taken together, the results suggest that Grail deficiency might suppress intracellular de novo lipogenesis and lipid uptake, thereby preventing fatty acid metabolism in the liver cells.

### *GRAIL* overexpression aggravates lipid accumulation

To further verify the effect of Grail on the lipid accumulation, we established stable overexpression of *GRAIL* in the HepG2 and AML12 cells and measured the Grail levels (Fig. 4G, H). The mRNA levels of lipid metabolism-related genes significantly increased in the PA-treated HepG2/Grail compared to the controls (Fig. 4I). Similar observations were reported in the AML12/Grail cells (Fig. S3B). The protein levels of FASN, CD36, and the mature form of SREBP-1c increased in the PA-treated HepG2/Grail or AML12/Grail cells (Fig. 4G, H). Oil Red O staining results showed the apparent lipid accumulation in the HepG2/Grail cells compared to the HepG2/vector cells, as well as in THLE-2/Grail, Huh7/Grail and AML12/Grail cells (Fig. 4J–4L and S5). Collectively, Grail could augment intracellular lipid accumulation in the liver cells.

### Grail interacts with Sirt1 and promotes its ubiquitination

**Fig. 5:**
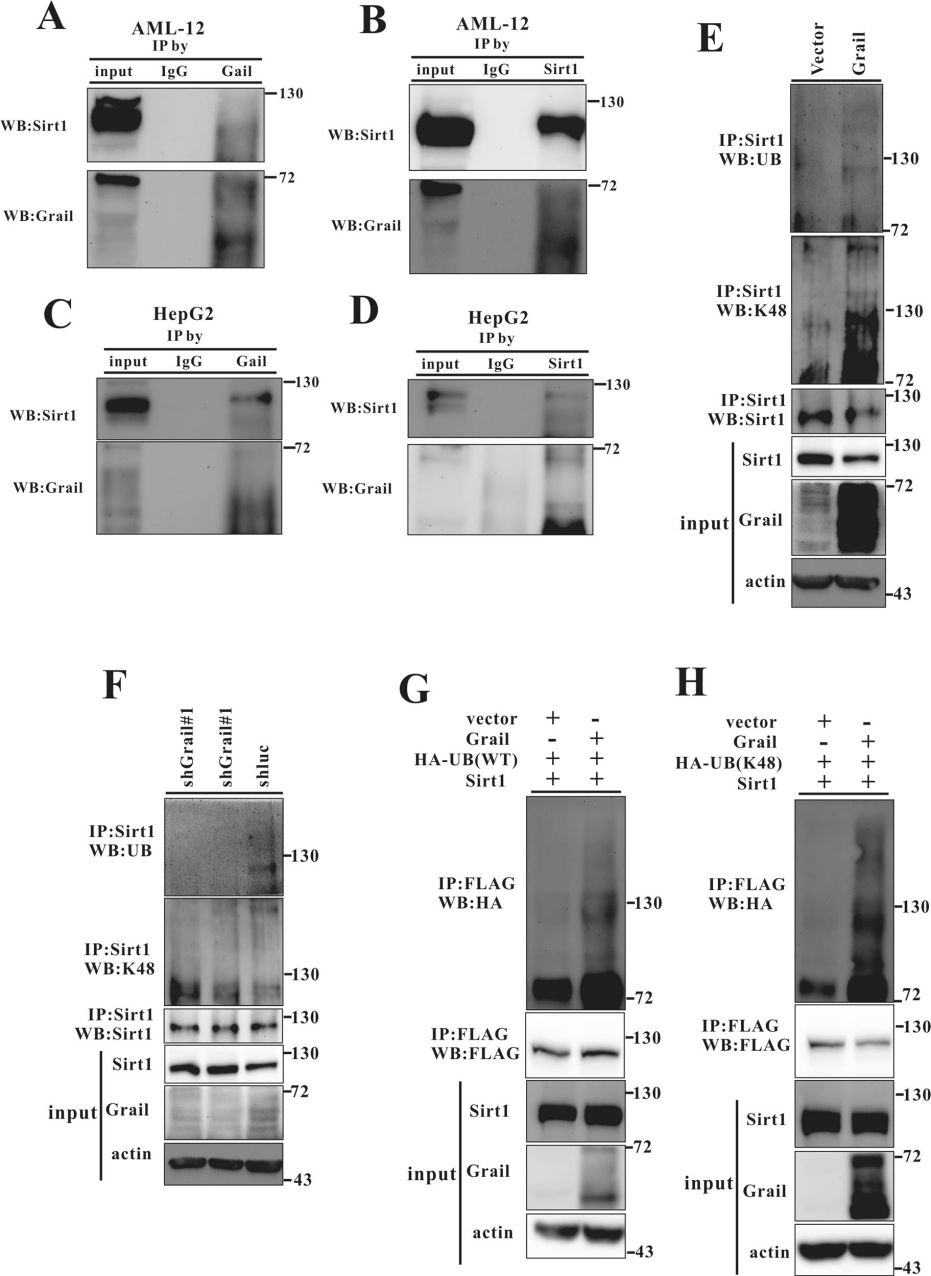
GRAIL interacts with sirtuin 1 (Sirt1) and enhances its ubiquitination. **A–D** Endogenous Grail interacts with endogenous Sirt1. Extracts from AML12 and HepG2 cells were prepared, immunoprecipitated with anti-Grail, anti-Sirt1, or rabbit anti-immunoglobulin G (anti-IgG) antibodies, and analyzed using anti-Grail and anti-Sirt1 antibodies. **E, F** Lysates from HepG2 (*GRAIL* overexpression or silencing) stable cell lines were harvested and subjected to immunoprecipitation (IP) with Sirt1 antibody and analyzed for ubiquitylation by immunoblotting with indicated antibody. **G, H** HEK293 cells were transiently transfected with HA-Ubiquitin (Ub) (wild-type (WT)), HA-Ub (K48), Flag-Sirt1, and Grail after 24 h. Lysates were harvested and subjected to IP with Flag antibody and analyzed for ubiquitylation by immunoblotting with indicated antibody.

Previous studies have documented that Sirt1 through its involvement in the regulation of lipid metabolism, plays a key role in NAFLD progression^21–23^. In order to examine whether Grail interacts with Sirt1 to exacerbate lipid accumulation, we performed co-immunoprecipitation (Co-IP) assay with HepG2 or AML12 cells. A reciprocal interaction between Grail and Sirt1 was observed in the HepG2 and AML12 cells (Fig. 5A–D). Since Grail is an E3 ubiquitin ligase that enhances the ubiquitylation of target proteins^18,24,25^, we suggested that Grail directly promotes Sirt1 ubiquitination. To verify the same, we transfected the HEK293 cells with plasmids encoding Grail, ubiquitin (Ub) and Sirt1, and analyzed their expressions. As shown in Fig. 5G, the co-expression of Grail with Sirt1 significantly elevated Sirt1 ubiquitination. Examining the ubiquitination of the endogenous Sirt1 in the cells, we observed that *GRAIL* overexpression promoted Sirt1 ubiquitination compared to the controls (Fig. 5E). On the contrary, *GRAIL* knockdown cells exhibited apparent decrease in the Sirt1 ubiquitination (Fig. 5F). To further investigate Grail-mediated Sirt1 polyubiquitination, we used mutant Ub containing a single lysine (K48). Grail-mediated polyubiquitination of Sirt1 was significantly increased in the presence of hemagglutinin (HA)-Ub-K48 (Fig. 5H). An apparent increase in the K48-ubiquitination of Sirt1 was observed in the HepG2 cells overexpressing *GRAIL* (Fig. 5E). The observation was reversed with silencing of Grail (Fig. 5F). The reported data indicated that Grail mediates K48-linked ubiquitination of Sirt1.

**Fig. 6:**
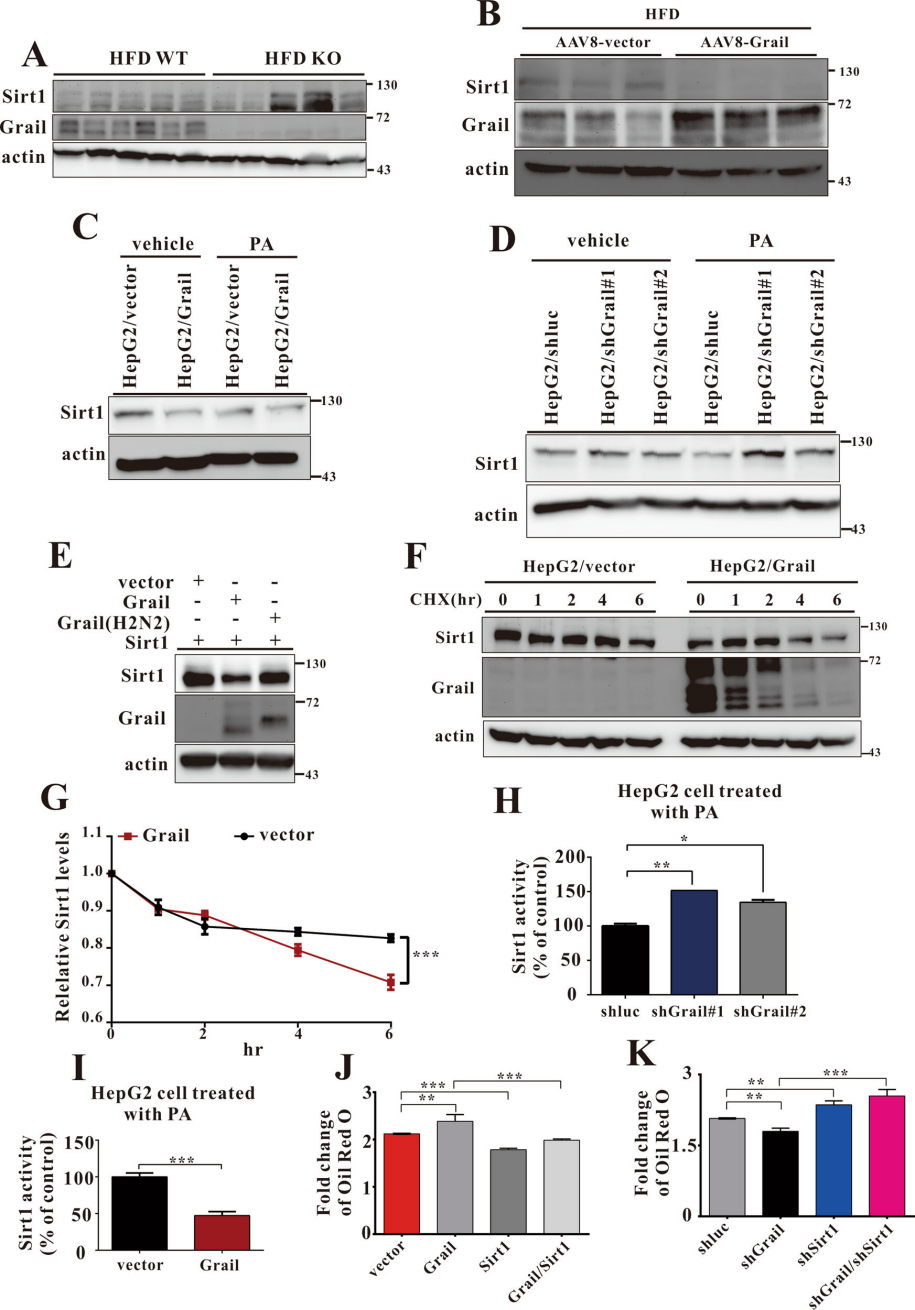
Grail influences sirtuin 1 (Sirt1) protein level and activity. **A**, **B** Immunoblotting measurement of Sirt1 expression in the liver samples obtained from wild-type (WT), Grail knockout (KO), AAV8-vector, and AAVV8-Grail mice fed high-fat diet (HFD) for 8 weeks. **C**, **D** Endogenous Sirt1 protein levels in indicated stable cell lines with or without palmitic acid (PA) treatment. **E** Effects of WT or mutant GRAIL overexpression on Sirt1 degradation. HEK293 cells were transfected with 0.5 μg of vector, Grail or Grail (H2N2) in the presence of 0.5 μg of pcDNA-flag-Sirt1. The cells were then harvested and subjected to immunoblotting. **F**, **G** HepG2/vector and HepG2/Grail cells were added with cycloheximide (CHX, 100 g/ml), and cells were harvested at the indicated times. Cells lysates were subjected to immunoblotting with the indicated antibodies. **H**, **I** Endogenous Sirt1 activity was measured in isolated nuclear extracts from HepG2 (GRAIL overexpression or silencing) stable cell lines. Sirt1 activity was expressed as a percentage of the control activity. **J**, **K** Oil Red O staining in the indicated HepG2 stable cell lines treated with PA for 24 h. The data are presented as mean ± SD. Student’s *t*-test are used to evaluate the statistical significance. **P < 0.01; ***P < 0.001.

### Grail reduces Sirt1 protein levels

On determining the possible regulation of Sirt1 by Grail, we observed that the Sirt1 levels were markedly increased in the livers derived from HFD-fed KO mice compared to the HFD-fed WT mice (Fig. 6A). Conversely, Sirt1 levels apparently decreased in the livers of HFD-fed AAV8-Grail mice compared to the HFD-fed AAV8-Vector mice (Fig. 6B). To further substantiate the Sirt1 regulatory effects of Grail, we evaluated the Sirt1 levels in *GRAIL*-overexpressing HepG2 cells. Congruent with the expectations, overexpression of *GRAIL* significantly reduced the levels of endogenous Sirt1 (Fig. 6C). Conversely, short hairpin RNA (shRNA)-mediated stable knockdown of *GRAIL* in HepG2 cells reversed the effect (Fig. 6D). While overexpression of *GRAIL* significantly reduced the levels of exogenous Sirt1, the RING finger domain mutant of Grail (H2N2) had no effect on the Sirt1 protein levels (Fig. 6E). We then used cycloheximide (CHX), a de novo protein synthesis inhibitor, to investigate whether decreased protein stability was responsible for the Grail-mediated downregulation of Sirt1 level. Congruent with the speculation, the observation substantiated that Grail influences the Sirt1 protein level by reducing its protein stability (Fig. 6F, G). The results indicated that the Sirt1 levels were affected by Grail-mediated degradation of Sirt1, which might contribute to the cellular outcome of Sirt1 in response to lipid metabolism.

**Fig. 7:**
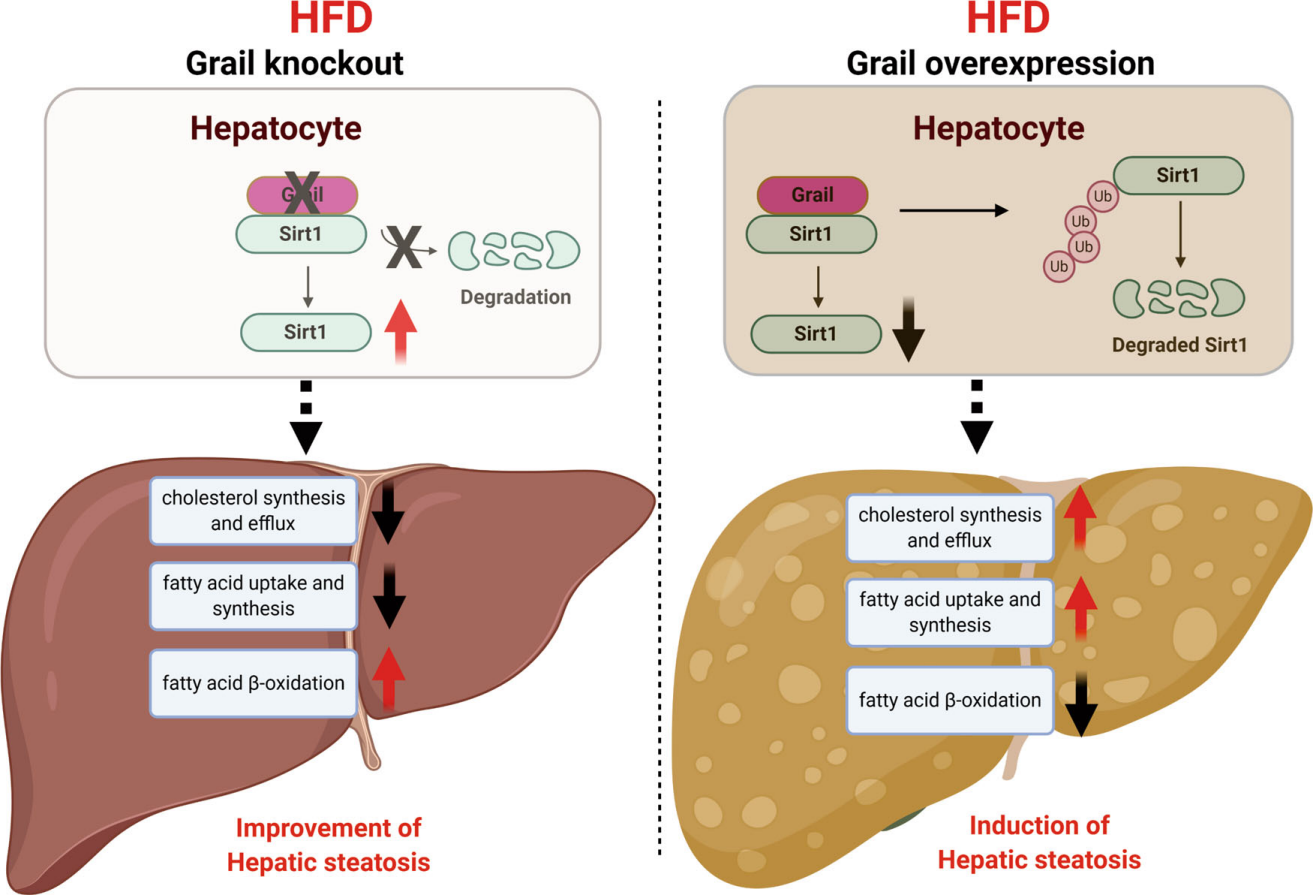
The working model of Grail in the regulation of hepatic steatosis. Grail expression is increased in hepatocytes with high-fat diet feeding. Grail interacts with sirtuin 1 (Sirt1) and subsequently degrades it by ubiquitination. This results in increased fatty acid uptake and synthesis and decreased beta-oxidation of fatty acid, thereby, synergistically aggravating fatty acid accumulation and hepatic steatosis.

### Grail inhibits endogenous Sirt1 activity

Several studies have showed altered Sirt1 activity during metabolic processes^26–28^. We therefore analyzed the role of Grail in controlling Sirt1 activity in the PA-treated HepG2 cells. As shown in Fig. 6H, a significant increase in the Sirt1 activity was observed in the HepG2/shGrail cells compared to the PA-treated control cells. Conversely, overexpression of *GRAIL* inhibited the Sirt1 activity in the PA-treated HepG2/Grail cells (Fig. 6I). In order to further validate that the Grail-mediated induction of lipid accumulation was facilitated through the inhibition of Sirt1, we overexpressed *GRAIL*, *SIRT1*, or both in HepG2 cells and thereby analyzed the lipid accumulation after PA treatment. While apparent lipid induction was observed upon *GRAIL* overexpression in PA-treated HepG2 cells, a comparative decrease was evident in the HepG2/Grail/Sirt1 cell lines (Fig. 6J). Interestingly, decreasing lipid accumulation was restored in HepG2/shGrail cells, upon Sirt1 silencing (Fig. 6K). Hence, our data suggested that Grail-mediated regulation of Sirt1 level and activity might play an important role in the progression of hepatic steatosis.

### Grail colocalizes with Sirt1 in the nucleus or cytoplasm

Finally, using immunofluorescence microscopy, we examined the subcellular localization of endogenous Grail and Sirt1 proteins in PA-treated HepG2 cells. While PA reduced the accumulation of Sirt1 proteins, the level of nuclear Grail protein increased (Fig. S6). We also found that colocalization of Grail with Sirt1 was either in the nucleus or in the cytoplasm (Fig. S6). Confocal imaging of the HepG2 cells also yielded the same results (Fig. S7). We further silenced endogenous *GRAIL* expression and found that endogenous Sirt1 proteins were induced. On the contrary, overexpression of *GRAIL* resulted in reduced levels of endogenous Sirt1 protein (Figs. S8-9). The findings were consistent with our previous data that suggested Grail interacts with Sirt1 and is involved in its stability.

## Discussion

Liver is an important organ for maintaining the homeostasis of the human body. Loss of liver function might result in reduced nutrient availability and lack of energy recycling ability. Obesity or metabolic syndrome-induced NAFLD involving excessive cytoplasmic accumulation of triglyceride, poses as the most common liver disease. NAFLD stimulates hepatic inflammation and NASH, and finally leads to hepatocellular carcinoma. In this study, we observed an apparent induction in *GRAIL* expression in the human and mice liver samples with NAFLD; Grail could affect the cellular lipid accumulation in hepatocytes, in vitro and in vivo. The liver injury index was reduced in *GRAIL* KO mice and elevated in the *GRAIL*-overexpressed mice. Since Grail is an E3 ligase protein, it does not remain associated with direct transcriptional regulation of target proteins. Thus, we searched for factors regulated by Grail. Here, we revealed Sirt1 as one of the targets of Grail. Grail not only interacts with Sirt1 but also modulates its expression level and enzyme activity (Fig. 7). Although Sirt1 regulates multiple transcription factors and cofactors involved in metabolism, it is yet unknown that whether Sirt1 and Grail interaction influences the Sirt1 interacting network. This indeed poses as an interacting question following the interaction of Grail with Sirt1.

To further expand the scope of the previous study on molecular basis of obesity, we examined the role of Grail in hepatic steatosis in an HFD-induced hepatic steatosis model. Observations revealed a considerably low level of hepatic lipid accumulation in HFD-fed Grail KO mice, owing to the suppression of the genes involved in the lipid synthesis and uptake, and increased expression of the fatty acid β-oxidation-related genes. Conversely, the results were reversed after HFD feeding in *GRAIL* KO mice with AAV8-mediated overexpression of *GRAIL*. While the *SIRT1* expression was significantly reduced in the liver of *GRAIL* KO mice after HFD feeding, the same was upregulated in the liver of HFD-fed AAV8-Grail mice. This suggests that Grail aggravates the progression of hepatic steatosis by inhibiting the function of Sirt1. Considering the notion that the functional mitochondria contributes towards the homeostasis of fatty acid oxidation, in accordance with the study findings it is also likely that Grail potentially influences the function of mitochondria in a Sirt1-dependent pathway. Future studies are thus warranted for a detailed molecular insight into the Grail-mediated regulation of the mitochondrial function.

Sirtuin family of proteins shares a conserved catalytic domain and a nicotinamide adenine dinucleotide (NAD+) binding region. Sirt1 belonging to the sirtuin family also contains a conserved NAD+-dependent deacetylase enzyme. Till date, seven sirtuin members (Sirt1-7) have been discovered. Sirt1, Sirt6, and Sirt7 remains localized in the nucleus and controls metabolism. Sirt3, Sirt4, and Sirt5 are distributed within the mitochondria and affects the metabolic pathways as well. It is likely, that Sirt1 among the sirtuin family of proteins may not exclusively interact with Grail. Further investigations are required to clarify this possibility.

Downregulation of Grail can reduce lipid accumulation and inhibit the expression of genes associated with lipogenesis after PA treatment or HFD feeding. Expression of a part of genes involved in β-oxidation, fatty acid synthesis and uptake, and cholesterol synthesis and efflux were lower in the *GRAIL* KO mice than WT mice subjected to ND feeding. Grail may affect these metabolic enzymes through direct or indirect pathways. To further build Grail-regulated networks, future studies should consider employing next-generation sequencing (NGS) technique.

In conclusion, our study identified Grail as a potential regulator of hepatic steatosis. The underlying mechanism was also elucidated. We believe that the study findings would provide new insights into the development of NAFLD treatment strategies.

## Experimental procedures

### Animals experiments

All animal experiments were approved by the National Defense-Medical Center Animal Experiment Ethics Committee (IACUC-19-021). The *GRAIL* KO mice were prepared as per the previous study^18^. The mice were housed under a regular 12-h light/ dark cycle for 2 weeks before the initiation of the experiments. Eight-week-old male WT and *GRAIL* KO mice were fed ad libitum with either a ND or HFD (D12492; 60 kcal% fat, 20 kcal% protein, 20 kcal% carbohydrate; Research Diets, New Brunswick, NJ), for 8 weeks. The mice were kept on a 12-h light/dark cycle at 22 ± 1 °C.

### In vivo administration of AAV

AAV8-Vector and AAV8-Grail were administered by tail vein injection at a dose of 1 × 10^12^ vg/mice in a total volume of 200 µl into 7-week-old male C57BL/6J mice. One week after treatment feeding with a 60% HFD (D12492) diet and were maintained for 8 weeks. The mice were kept on a 12-h light/dark cycle at 22 ± 1 °C.

### Cells, plasmids, and transfection procedures

HepG2 cells were cultured in Dulbecco’s modified Eagle’s medium (DMEM) supplemented with 10% fetal bovine serum (FBS). AML12 cells were grown in a 1:1 mixture of DMEM high glucose and Ham’s F-12, supplemented with 10% FBS. The immortalized human liver cell line, THLE-2 was cultured using the bronchial epithelial cell growth medium (BEGM) Bullet Kit (Lonza, Basel, Switzerland). To mimic HFD condition, cells were grown to a confluency of 70–80% and treated with 800 μM palmitic acid in DMEM containing 10% FBS. The *GRAIL* was cloned into pCMV-TNT vector (Promega, CA, USA) using the EcoRI and BamHI sites. The Flag-SIRT1 plasmid was purchased from Addgene (Addgene, Watertown, Massachusetts, USA). Transfection was performed using jetPRIME (New York, USA), according to the manufacturer’s instructions. Cells were plated at a low density (~1 × 10^5^ cells/60 mm dish) and allowed to grow up to 50–60% confluence. Thereafter, jetPRIME-mediated gene transfection was performed and the resulting transfected cells were lysed in the radioimmunoprecipitation assay (RIPA) buffer.

### Primary hepatocyte isolation and culture

Primary hepatocytes were isolated from WT or *GRAIL* KO mice subjected to ND or HFD feeding. The liver was perfused via the inferior vena cava cannula at outflow of perfusate. The liver was first perfused with 100 ml of phosphate-buffered saline (PBS) with 0.5 mM ethylenediaminetetraacetic acid (EDTA) and then with 25 ml of PBS, 0.5 mM EDTA, and 0.1% of collagenase Type IV (Sigma–Aldrich, St. Louis, Missouri, USA). All solutions were brought to room temperature before use. The liver lobes were transferred to a culture dish containing William’s E Medium (WEM, GIBCO, Thermo Fisher Scientific, Waltham Massachusetts, USA) and teared into pieces with forceps. The hepatic cells were filtered with 70 um filters into 50 ml tubes and centrifuged at 50 × *g* for 3 min at 4 °C. The hepatocyte pellet was then washed twice with WEM. Finally, the hepatocytes were resuspended in WEM containing 10% FBS. After incubation at 37 °C with 5% carbon dioxide (CO_2_) for 3 h, the hepatocytes were washed to remove dead cells and fresh culture medium was added.

### Immunoprecipitation and immunoblotting

The proteins were recovered from the cells after being harvested in the lysis buffer (50 mM Tris, pH 8.0, 5 mM sodium chloride (NaCl), 0.5% NP-40, and 1× protease inhibitor) and freeze/thawed for three times. Protein concentration was determined using the Bradford method (Bio-Rad, CA, USA). Cell extracts containing equivalent amounts of protein were immunoprecipitated overnight at 4 °C in lysis buffer containing a polyclonal antibody against Grail or Sirt1. Dynabeads Protein G (Invitrogen, Carlsbad, California, USA) was added to the immunoprecipitation mixture and incubated for 1 h at 4 °C. Thereafter, the samples were washed using SNNTE buffer (5% sucrose, 1% NP-40, 0.5 M NaCl, 50 mM Tris, pH 7.4, and 5 mM EDTA). The immunoprecipitates were resuspended in sodium dodecyl sulfate-polyacrylamide gel electrophoresis (SDS-PAGE) sample buffer, boiled, and loaded onto an SDS- polyacrylamide gel. After electrophoresis, the gel was transferred onto a nitrocellulose membrane and the blot was probed with the indicated primary antibodies. Proteins of interest were detected using enhanced chemiluminescence reagents (GE Healthcare, Chicago, Illinois, USA). The primary antibodies used for immunoprecipitation were anti-Grail, anti-Sirt1, and anti-Flag antibodies. The primary antibodies used for immunoblotting were: anti-Sirt1 (Millipore, Burlington, Massachusetts, USA), anti-FASN (Cell Signaling Technology, Danvers, Massachusetts, USA), anti-CD36 (Cell Signaling Technology), anti-SREBP-1c (Santa Cruz Biotechnology Inc., Dallas, Texas, USA), anti-Ub (Cell Signaling Technology), anti-k48Ub (Cell Signaling Technology), anti-actin (Chemicon International, Temecula, California, USA), and anti-Grail antibodies.

### In vivo ubiquitination assays

The HepG2/Grail or HepG2/shGrail cells were lysed in lysis buffer (50 mM Tris pH 8.0, 5 mM NaCl, 0.5% NP-40, and 1× protease inhibitor), freeze/thawed three times, and the proteins were recovered. Cell extracts containing equivalent amounts of protein were immunoprecipitated overnight at 4 °C in lysis buffer containing antibody against Sirt1. Dynabeads Protein G (Invitrogen) was added to the immunoprecipitation mixture and incubated for 1 h at 4 °C. Thereafter, the samples were washed thrice with SNNTE buffer (5% sucrose, 1% NP-40, 0.5 M NaCl, 50 mM Tris, pH 7.4, and 5 mM EDTA). The immunoprecipitates were then resuspended in SDS-PAGE sample buffer and loaded onto the SDS- polyacrylamide gel. After electrophoresis, the gel was transferred onto a nitrocellulose membrane and the blot was probed with the indicated primary antibodies. Proteins of interest were detected using enhanced chemiluminescence reagents (GE Healthcare).

### Virus particle production, viral transduction, and RNA interference

*GRAIL* or *SIRT1* was cloned into vector pQCXIP (Clontech Laboratories, Mount View, California, USA). The pQCXIP-Grail, pQCXIP-Sirt1, and pQCXIP-empty plasmids were transfected into GP2-293 cells using jetPRIME (New York, USA). The shRNA oligonucleotides were cloned into the expression vector, pSIREN-Retro-Q (Clontech Laboratories). The retroviruses were prepared according to the protocol available at the Clontech website. (Grail shRNA target sequence 1: 5′*-GAGGCATCCAAGTCACAATGG-3*′; Grail shRNA target sequence 2: *5*′*-GCAGGAAGCAGAGGCAGTTAA-3*′; Sirt1 shRNA target sequence 1: *5*′*-CAGGTCAAGGGATGGTATT-3*′). Cells were infected with the indicated retroviruses in the selection medium containing 2 μg/mL polybrene. After 48 h from infection, the cells were treated with 8 μg/mL puromycin to select for the pool of puromycin-resistant clones. The AAV expression vector was constructed and manipulated using the Helper Free Expression System (Cell Biolabs, Inc., San Diego, California, USA). *GRAIL* was cloned into the AAV expression vector, pAAV-MCS. AAV8 overexpressing *GRAIL* was generated according to standard protocols.

### Biochemical analysis

The serum TG, cholesterol, ALT, and AST were measured using commercially available kits (FUJIFILM: TG-P III, TCHO-P III, GPT/ALT-P III, and GOT/AST-P III).

### Real-time quantitative polymerase chain reaction (RT-qPCR)

RNAs from cells and tissues were isolated using the TRIzol reagent (Sigma–Aldrich). Complementary DNA (cDNA) was synthesized using Epicentre MMLV (Lucigen, Middleton, Wisconsin, USA). Cellular gene expression was analyzed using Applied Biosystems 7500 Real-Time PCR system and the IQ2 FAST Q-PCR kit. Gene expression in the tissues was determined using a Roche LightCycler 480. The primers used are listed in Supplementary Table 1.

### Immunohistochemistry and human liver samples

Human normal and nonalcoholic fatty liver samples were purchased from Raya Biotech (Raya Biotech, Taipei, Taiwan). Liver tissues were fixed overnight in 4% formaldehyde at room temperature and then embedded in paraffin. The sections were stained with Grail antibody or by HE staining. The frozen liver sections were subjected to oil red O staining for observing the lipid droplets in the liver (Sigma–Aldrich).

### Oil Red O staining

The cells were fixed in 10% formaldehyde, washed twice with 60% isopropanol and stained with oil red O stain. The cellular oil red O was eluted with 100% isopropanol and the absorbance was measured at 510 nm.

### Sirt1 activity in cells

The Sirt1 activity was determined with Sirt1 Activity Assay Kit (Abcam, Cambridge, UK) according to the manufacturer’s protocol. Briefly, the cell lysates were incubated with fluoro-substrate peptide (0.2 mM), developer, and NAD (2 mM) for 30 min at 37 °C. The activity was determined on a fluorometric plate reader at an excitation wavelength of 360 nm and an emission wavelength of 460 nm.

### Statistical analysis

The graphing and statistical analysis of the data were performed using GraphPad Prism 7 (GraphPad Software). All data were expressed as mean ± standard deviation (SD). For comparison of multiple datasets, one-way analysis of variance (ANOVA) with multiple comparative analysis was used. For analysis of two datasets, an unpaired two-tailed Student’s *t-*test was used. *P* < 0.05 was considered statistically significant.

## Supplementary information

Supplementary information
