# Supplementary material for: E3 ubiquitin ligase Grail promotes hepatic steatosis through Sirt1 inhibition
Source: Cell Death Dis. 2021 Mar 26;12(4):323. doi: 10.1038/s41419-021-03608-9 (PMC7997893; doi:10.1038/s41419-021-03608-9)
Supplement: Supplementary file 1 — Supplementary information [file 41419_2021_3608_MOESM1_ESM.docx]

**Supplementary Information**

**Supplementary Figure Legends**

**
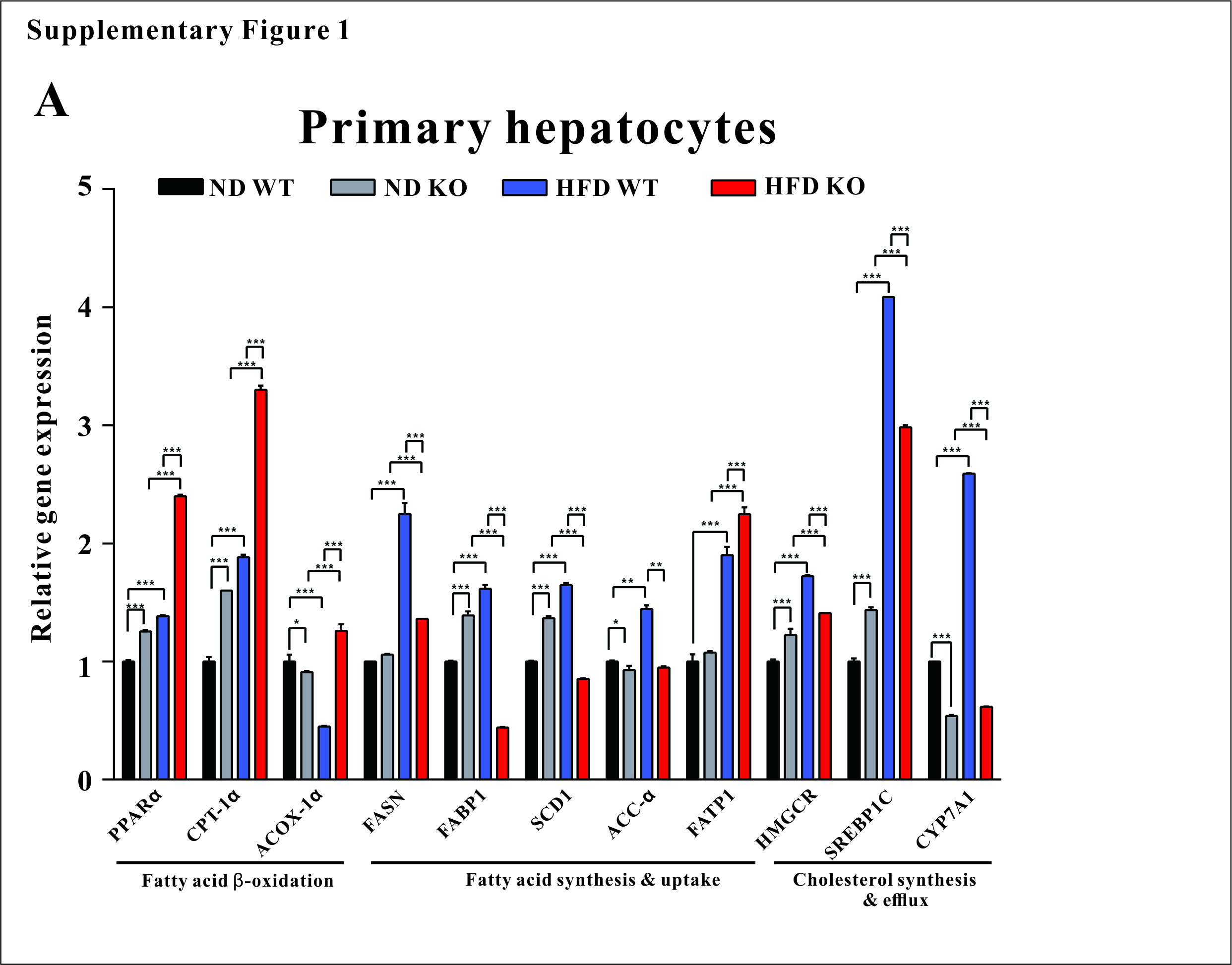
**

**Supplementary Fig. 1 Grail deﬁciency regulates the lipid metabolism-related genes expressions.** The mRNA expressions of genes related to cholesterol synthesis and efﬂux and fatty acid uptake, synthesis and b-oxidation were determined in the liver samples of WT and Grail KO mice after ND or HFD treatment. The data are presented as mean values ± SD. ^*^*P*<0.05; ^**^*P*<0.01; ^***^*P*<0.001, Student’s *t-*test


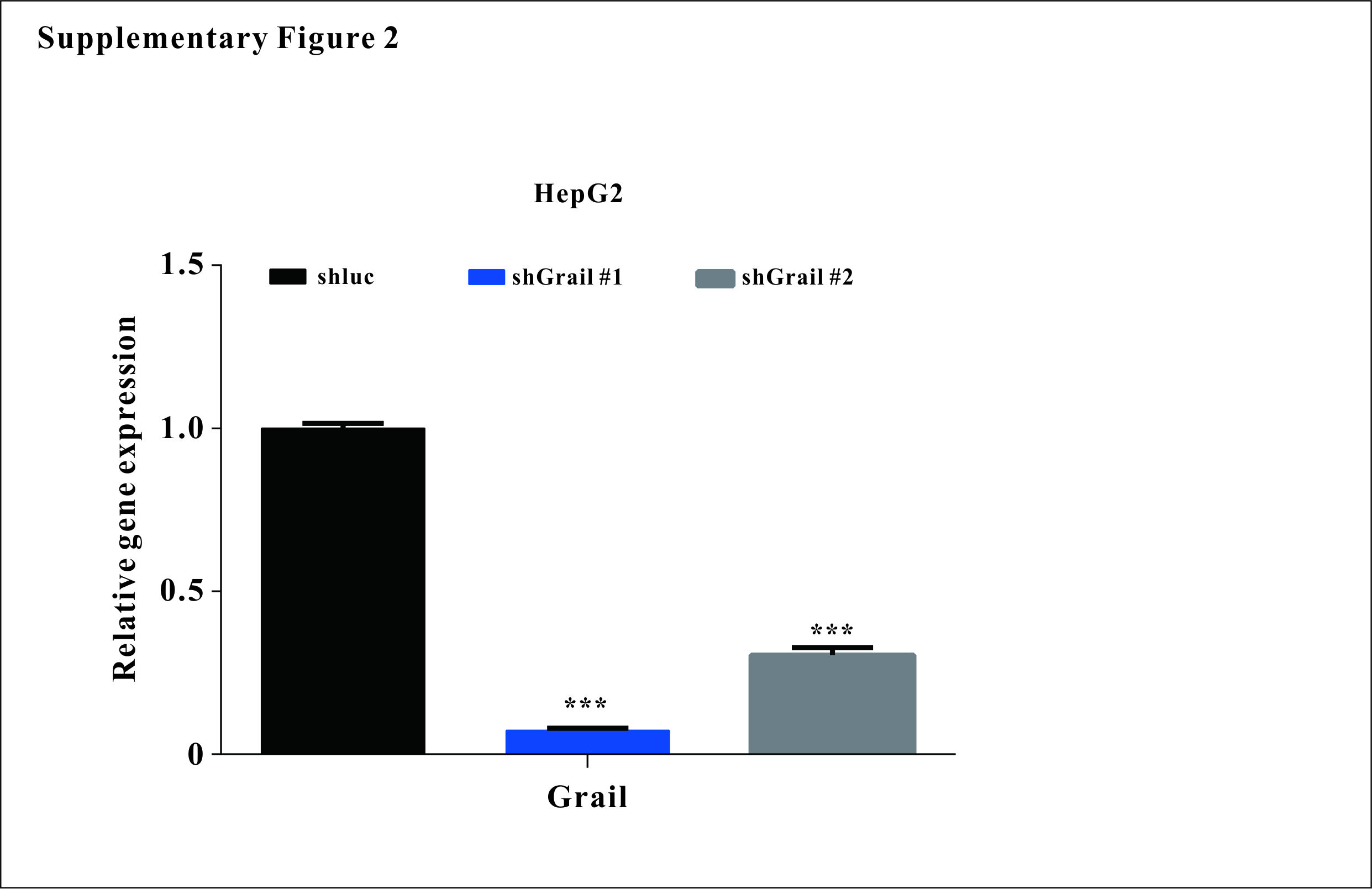


**Supplementary Fig. 2 The Grail mRNA expression level in HepG2/shGrail cell lines.** To measure mRNA expressions of Grail in HepG2/shGrail cell lines. The data are presented as mean values ± SD. ^***^*P*<0.001, Student’s *t-*test


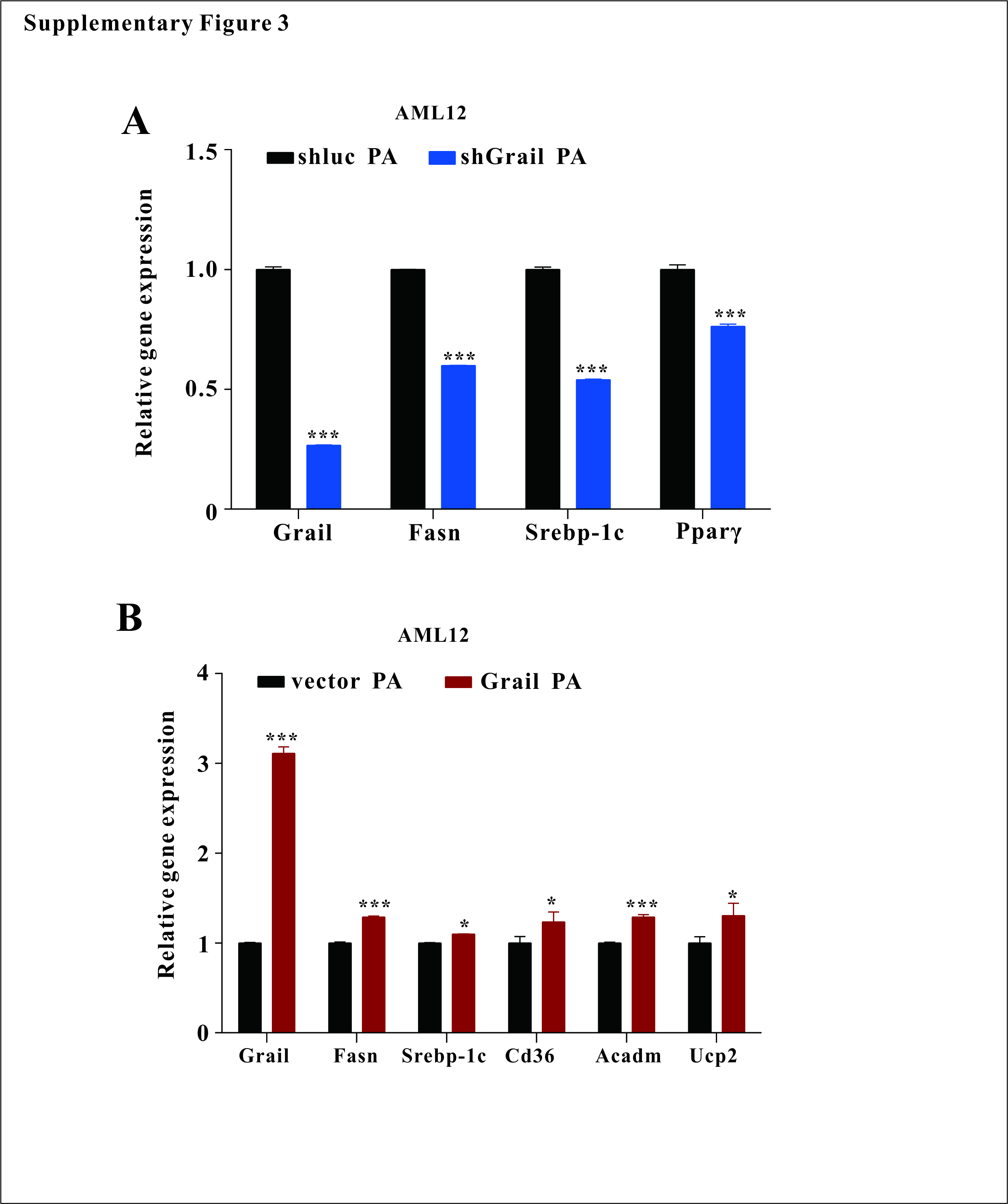


**Supplementary Fig. 3 The effects of Grail overexpression or silencing on the lipid metabolism-related genes expressions.** To measure mRNA expressions of genes related to lipid metabolism in (A) AML12/shGrail or (B) AML12/Grail cell lines, and subjected to PA (800 μM) for 24h.. The data are presented as mean values ± SD. ^*^*P*<0.05; ^***^*P*<0.001, Student’s *t-*test

**
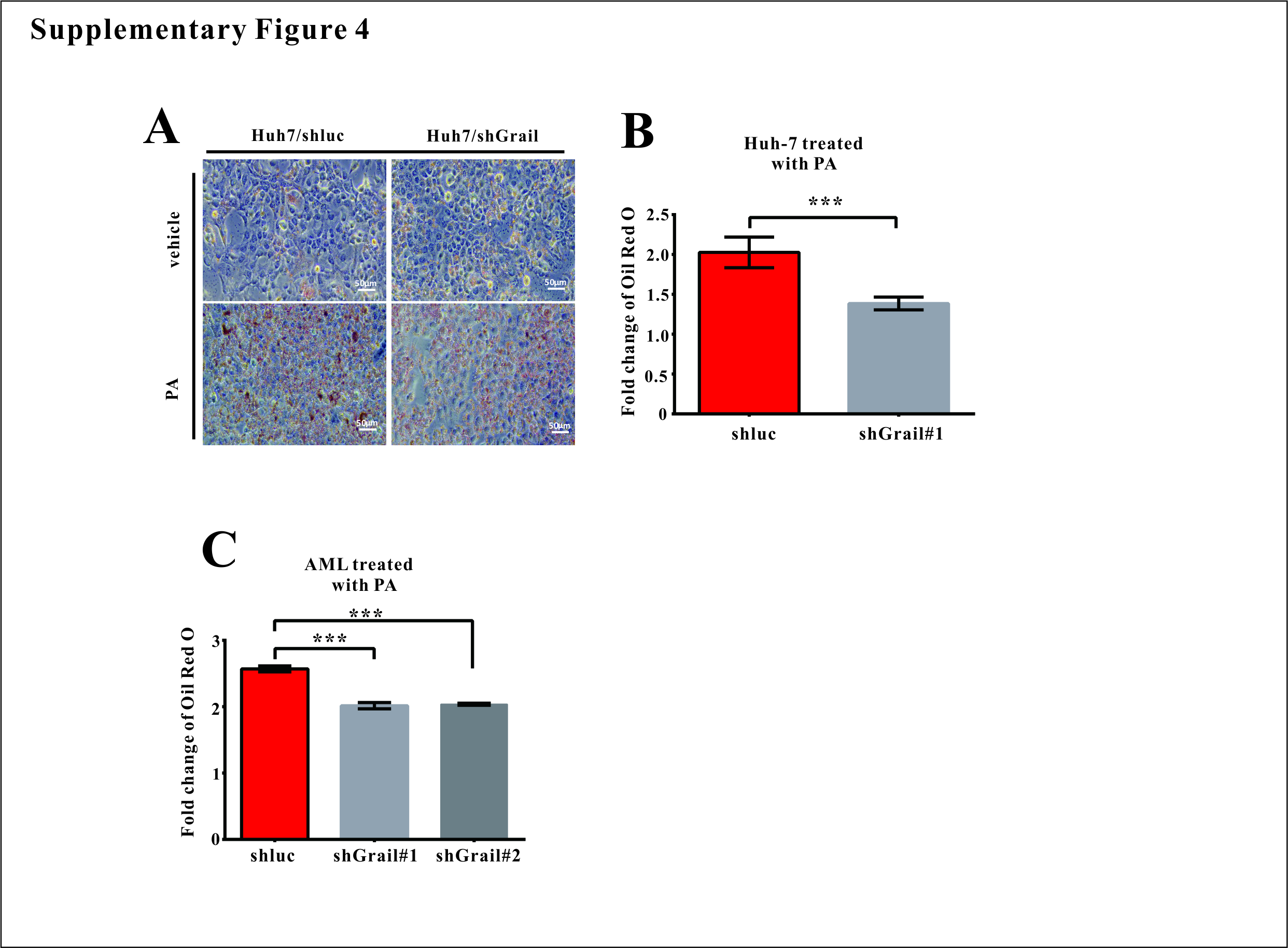
**

**Supplementary Fig. 4 The effects of Grail silencing on the lipid accumulation.** (A-B) Lipid accumulations displayed by Oil red O staining in Huh7/shluc and Huh7/shGrail or (C) inAML12/shluc and AML12/shGrail cell lines, and subjected to PA (800 μM) or vehicle control administration for 24h. The data are presented as mean values ± SD. ^***^*P*<0.001, Student’s *t-*test


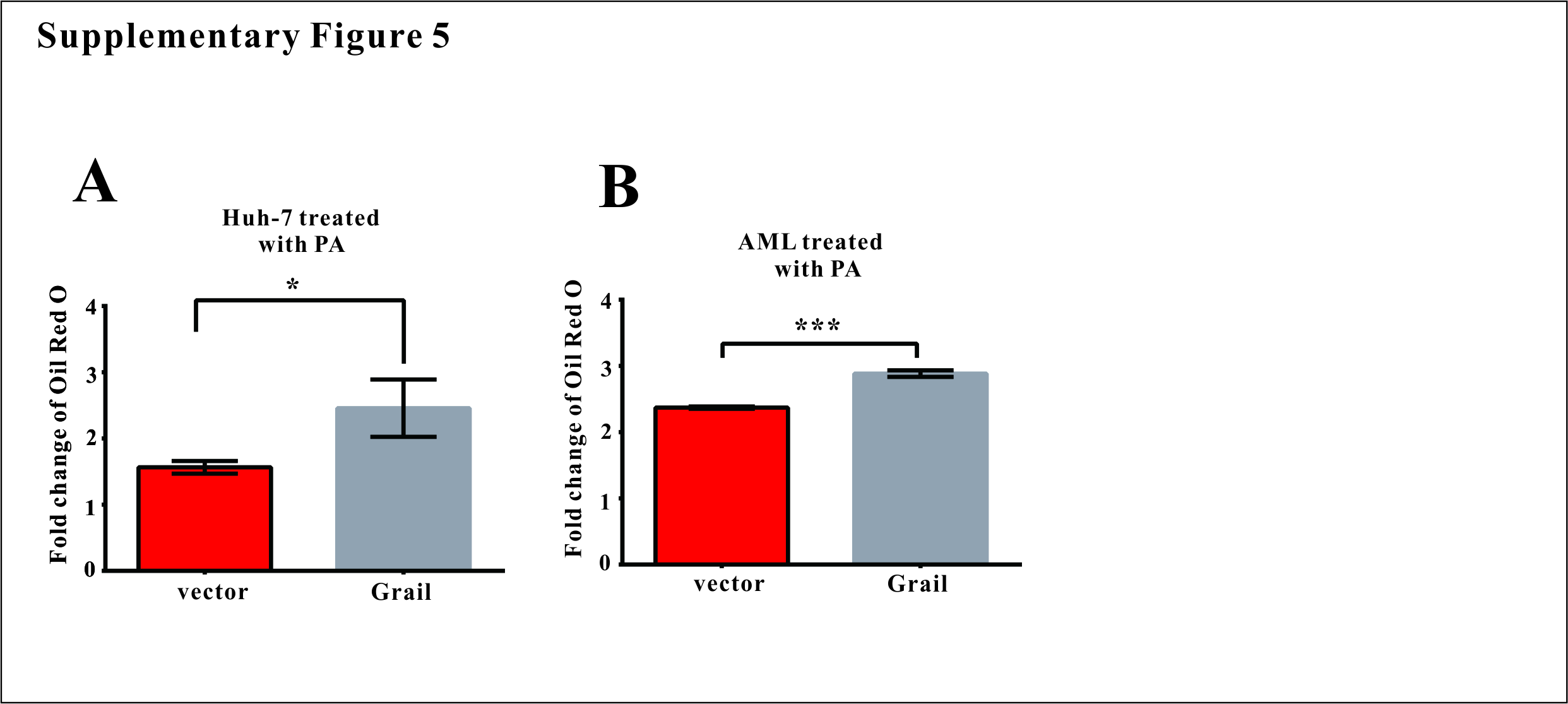


**Supplementary Fig. 5 The effects of Grail overexpression on the lipid accumulation.** (A) Lipid accumulations displayed by Oil red O staining in Huh7/vector and Huh7/Grail or (B) inAML12/vector and AML12/Grail cell lines, and subjected to PA (800 μM) or vehicle control administration for 24h. The data are presented as mean values ± SD. ^*^*P*<0.05;

^***^*P*<0.001, Student’s *t-*test


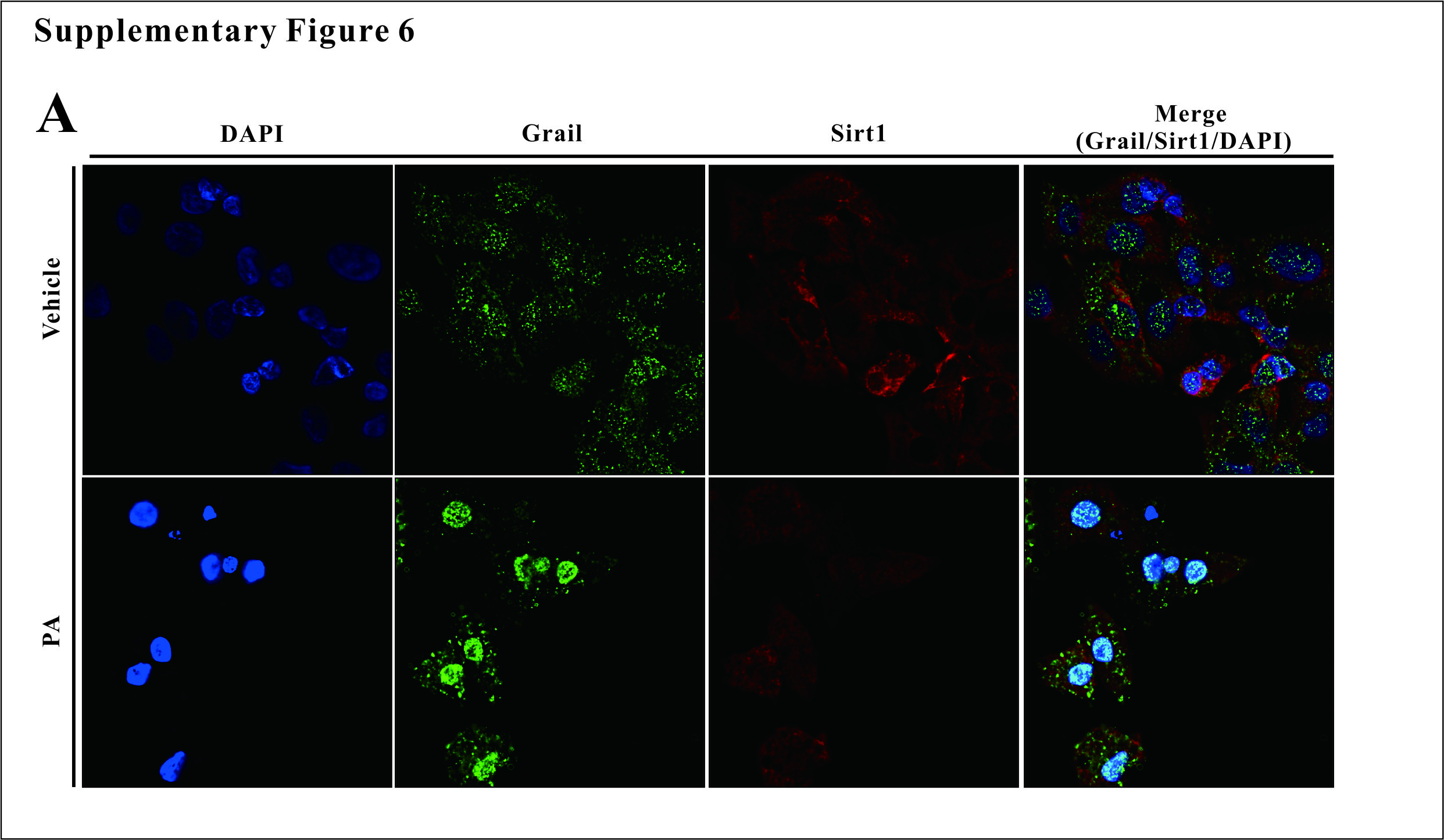


**Supplementary Fig. 6 Co-localization of Grail and Sirt1.** HepG2 cells were treated with PA (800 μM). After 24 hr, the subcellular localization of Grail and Sirt1 were examined by immunofluorescence microscopy (THUNDER Imaging Systems).


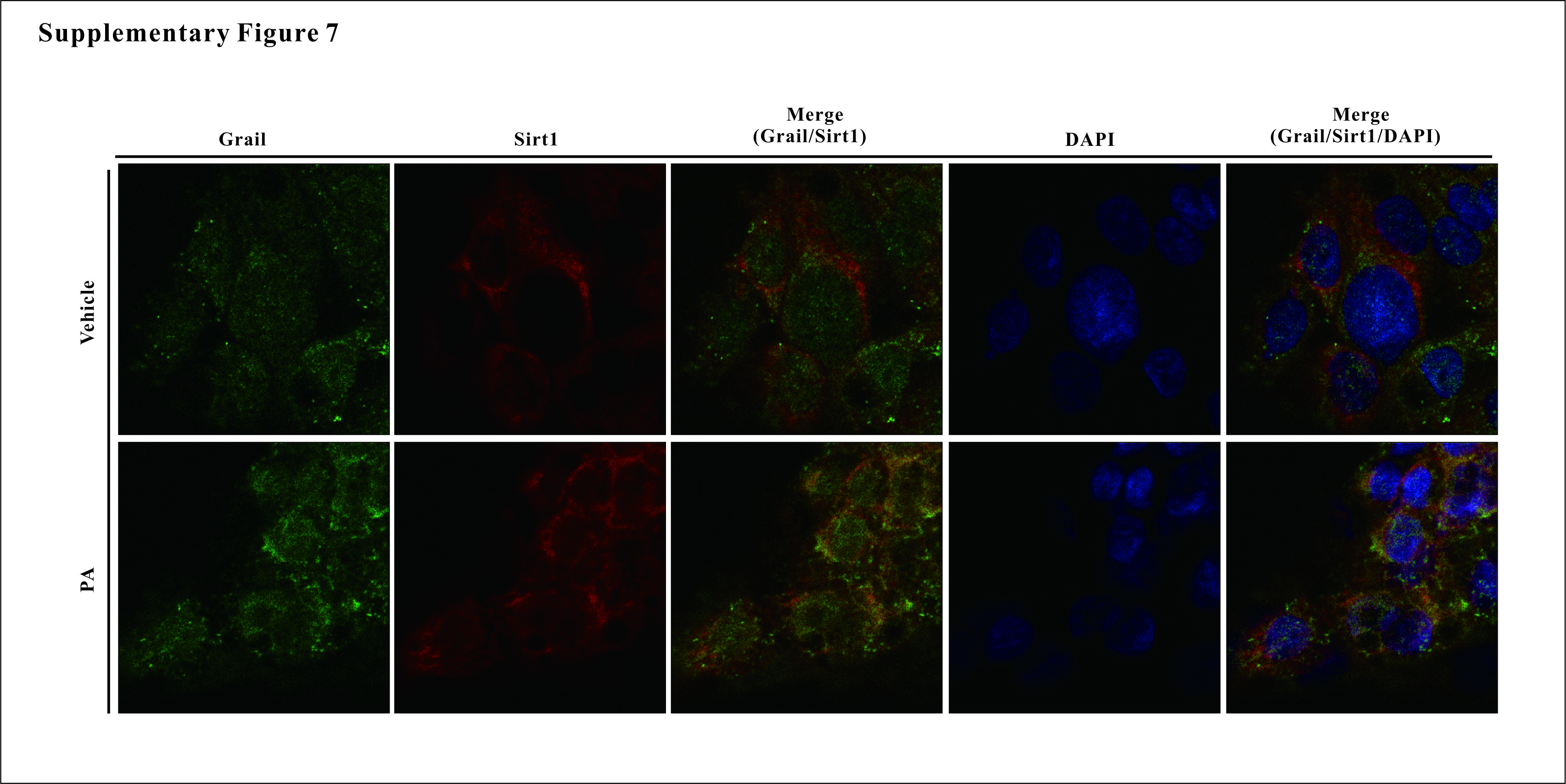


**Supplementary Fig. 7 Confocal fluorescence microscopy images of HepG2 cells treated with PA.** HepG2 cells were treated with PA (800 μM). After 24 hr, the subcellular localization of Grail and Sirt1 were examined by Confocal fluorescence microscopy (LSM880).

**
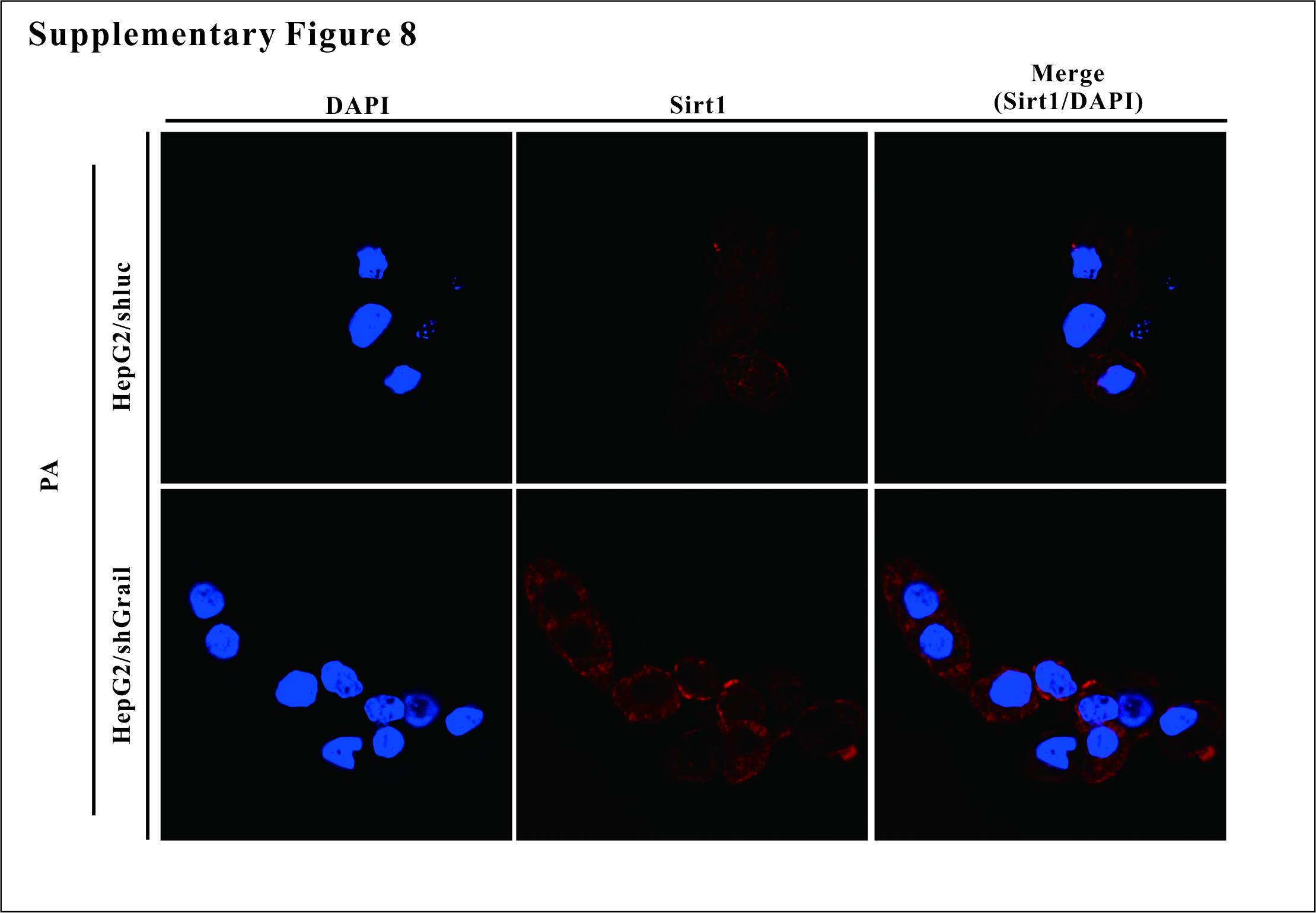
**

**Supplementary Fig. 8 Subcellular localization of Grail and Sirt1 in HepG2/shGrail cells.** HepG2 cells stably expressing the indicated shRNAs were treated with PA (800 μM). After 24 hr, the cellular localization of Grail and Sirt1 were examined by immunofluorescence microscopy (THUNDER Imaging Systems).

**
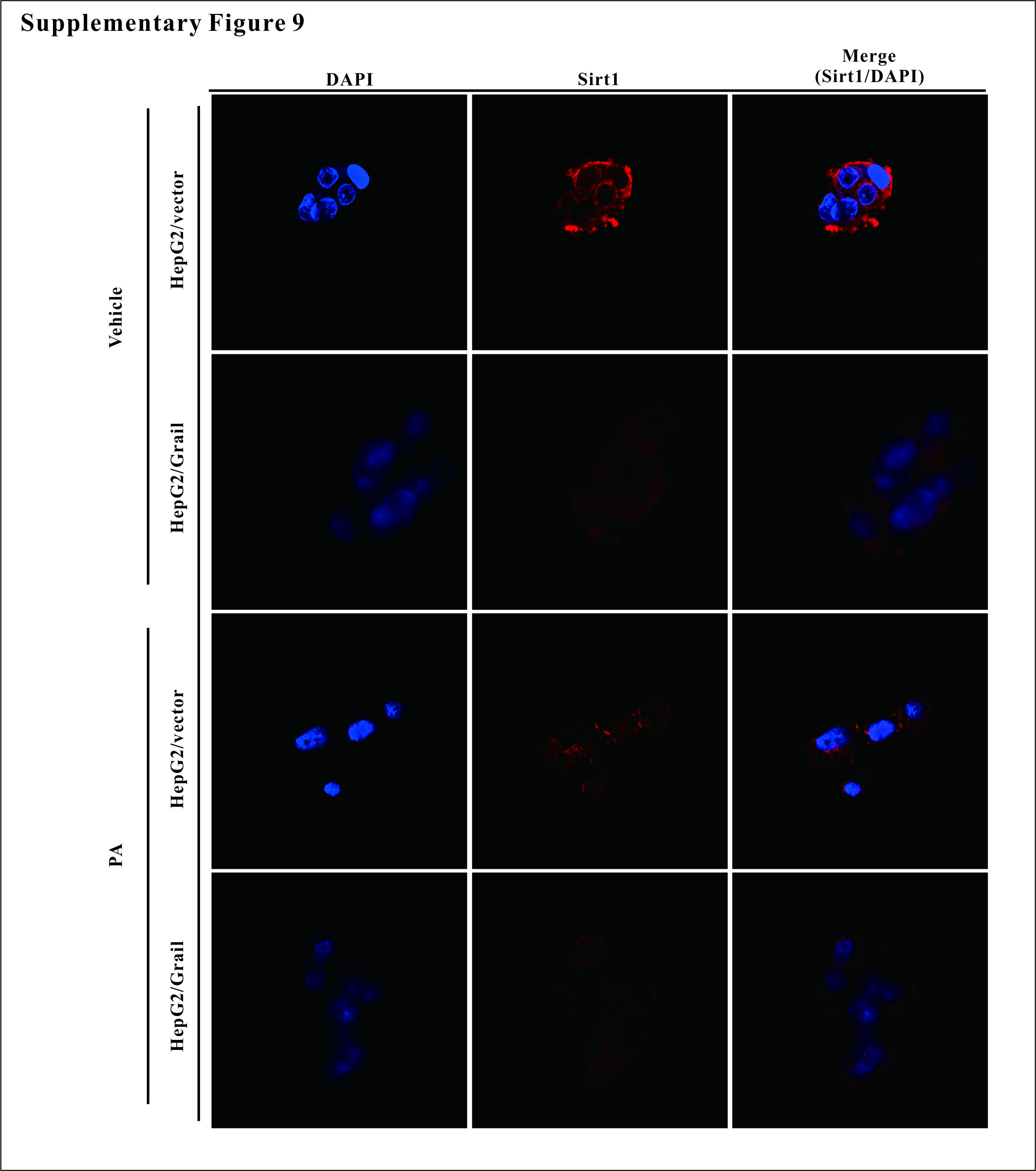
**

**Supplementary Fig. 9 Cellular images by immunofluorescenc microscopy of**

**HepG2/Grail cells.** HepG2 cells stably expressing Grail were treated with PA (800 μM). After 24 hr, the cellular localization of Grail and Sirt1 were examined by immunofluorescence microscopy (THUNDER Imaging Systems).

| **Supplementary Table 1 \| Primers used for Q-PCR analysis** | | |
| --- | --- | --- |
| **Primers** | **Forward sequence** | **Reverse sequence** |
| **Grail** | *5’-GCGTCTGGAGCCGTCATCTTTA-3’* | *5’-GGGCCATGTTTTTTCCCTACTTCTAT-3’* |
| **GAPDH** | *5’-TTCACCACCATGGAGAAGGC-3’* | *5’-GATGGCATGGACTGTGGTC-3’* |
| **Human FASN** | *5’-ACCTCCGTGCAGTTCTTGAG-3’* | *5’-GTTCAGGATGGTGGCGTAC-3’* |
| **Human SREBP-1c** | *5’-ATGGACGAGCCACCCTTCAG-3’* | *5’-AAGTGCAATCCATGGCTCCG-3’* |
| **Human FABP1** | *5’-GCACAGCAGTCAAAGCCATC-3’* | *5’-CGAGAACACACGTGGCAAAG-3’* |
| **Human ACC1** | *5’-CATCTCCCTTGGCCCAACC-3’* | *5’-TCTGAGCCAACAGAAGCAGG-3’* |
| **Human PPARα** | *5’-TCGGCGAGGATAGTTCTGG-3’* | *5’-GATAGCCTGAGGCCTTGTCC-3’* |
| **Human SCD1** | *5’-GGAGTCAGGGTGAACTGCAA-3’* | *5’-GGCTGTAGGGAATGCTGGTT-3’* |
| **Human CD36** | *5’-TCTCAATCTGGCTGTGGCAG-3’* | *5’-CAGGGTACGGAACCAAACTC-3’* |
| **Human HMGCR** | *5’-TGCAGCAAACATTGTCACCG-3’* | *5’-CTGCAAACAGGCTTGCTGAG-3’* |
| **Human CYP7A1** | *5’-AACTGGCAGAGAGCTTGAGG-3’* | *5’-ATGGTGTTTGCTTGCGATGC-3’* |
| **Human FATP1** | *5’-ATGGCTATGTCAGCGAGAGC-3’* | *5’-CTGGAACAGCCACCCCATAG-3’* |
| **Human DGAT1** | *5’-TATTGCGGCCAATGTCTTTGC-3’* | *5’-CACTGGAGTGATAGACTCAACCA-3’* |
| **Mouse FASN** | *5’-TCCACCTTTAAGTTGCCCTG-3’* | *5’-TCTGCTCTCGTCATGTCACC-3’* |
| **Mouse SREBP-1c** | *5’-TGAGGGTCAAAACCAGCCTC-3’* | *5’-CCGTCCACAAAGAAACGGTG-3’* |
| **Mouse FABP1** | *5’-GTCGTCCTACGCTACCTGTG-3’* | *5’-ACTGCCATAGAGCGCATACC-3’* |
| **Mouse ACC1** | *5’-GGCAGCAGTTACACCACATAC-3’* | *5’-ACACATGTCCGCCATCTTCC-3’* |
| **Mouse PPARα** | *5’-GAAAGACCAGCAACAACCCG-3’* | *5’-GGCAAATTCTGTGAGCTCCG-3’* |
| **Mouse SCD1** | *5’-CATGGCGTTCCAGAATGACG-3’* | *5’-AGCTTCTCGGCTTTCAGGTC-3’* |
| **Mouse CD36** | *5’-ATTGGCCAAGCTATTGCGAC-3’* | *5’-GCAAAG GCATTGGCTGGAAG-3’* |
| **Mouse CPT1α** | *5’-AAACCCACCAGGCTACAGTG-3’* | *5’-GTAATGTGCGAGCTGCAGTG-3’* |
| **Mouse HMGCR** | *5’-TTCTGGCAGTCAGTGGGAAC-3’* | *5’-TGACAATGTTTGCTGCGTGG-3’* |
| **Mouse CYP7A1** | *5’-CAAGACCGCACATAAAGCCC-3’* | *5’-GGTGTTTGCTTGAGATGCCC-3’* |
| **Mouse ACOX1α** | *5’-AGTGCTACGGGTTACATGCC-3’* | *5’-TTACATACGTGCCGTCAGGC-3’* |
| **Mouse FATP1** | *5’-TGGTTTCTGGGACTTCCGTG-3’* | *5’-AAGATGCACGGGATCGTGTC-3’* |
